# Maize network analysis revealed gene modules involved in development, nutrients utilization, metabolism, and stress response

**DOI:** 10.1186/s12870-017-1077-4

**Published:** 2017-08-01

**Authors:** Shisong Ma, Zehong Ding, Pinghua Li

**Affiliations:** 1 School of Life Sciences, University of Science and Technology of China, Hefei, Anhui China; 2 The Institute of Tropical Bioscience and Biotechnology, Chinese Academy of Tropical Agricultural Sciences, Haikou, Hainan China; 3 State Key Laboratory of Crop Biology, College of Agronomy, Shandong Agricultural University, Tai’an, Shandong China

**Keywords:** Comparative genomics, Gene network analysis, Maize development, Maize metabolism pathways, Plant nutrient uptake and utilization

## Abstract

**Background:**

The advent of big data in biology offers opportunities while poses challenges to derive biological insights. For maize, a large amount of publicly available transcriptome datasets have been generated but a comprehensive analysis is lacking.

**Results:**

We constructed a maize gene co-expression network based on the graphical Gaussian model, using massive RNA-seq data. The network, containing 20,269 genes, assembles into 964 gene modules that function in a variety of plant processes, such as cell organization, the development of inflorescences, ligules and kernels, the uptake and utilization of nutrients (e.g. nitrogen and phosphate), the metabolism of benzoxazionids, oxylipins, flavonoids, and wax, and the response to stresses. Among them, the inflorescences development module is enriched with domestication genes (like *ra1*, *ba1*, *gt1*, *tb1*, *tga1*) that control plant architecture and kernel structure, while multiple other modules relate to diverse agronomic traits. Contained within these modules are transcription factors acting as known or potential expression regulators for the genes within the same modules, suggesting them as candidate regulators for related biological processes. A comparison with an established Arabidopsis network revealed conserved gene association patterns for specific modules involved in cell organization, nutrients uptake & utilization, and metabolism. The analysis also identified significant divergences between the two species for modules that orchestrate developmental pathways.

**Conclusions:**

This network sheds light on how gene modules are organized between different species in the context of evolutionary divergence and highlights modules whose structure and gene content can provide important resources for maize gene functional studies with application potential.

**Electronic supplementary material:**

The online version of this article (doi:10.1186/s12870-017-1077-4) contains supplementary material, which is available to authorized users.

## Background

Advances over the past two decades have generated numerous transcriptome datasets. Increasingly, ever more complete transcriptome data can be merged and integrated with gene and genome structures. They provide unbiased snapshots of gene expression dynamics within organisms under various conditions. While cell-, organ-, or condition-specific expression profiles abound, it remains a key challenge to deduce the underlying gene regulatory circuits that control and give rise to the observed gene expression dynamics. To address this, gene network analysis has emerged as a tool that can filter and refine the analysis of large gene expression datasets. Such gene networks consist of genes (nodes) and connections (edges) between genes that represent co-expression dynamics or association patterns underlying the expression data. According to a ‘guilt-by-association’ paradigm, connected genes may have similar functions, be part of the same complex or pathway, or participate in the same signaling circuits [1]. Gene networks can assign putative functions to unknown genes based on functions revealed by their associates, or to identify novel genes for existing pathways [2, 3].

Different from random networks, gene networks generally identify gene modules categorizing and tracing groups of highly inter-connected genes that share similar expression patterns and, often, are recognizable by their relationship to a function in a particular biological process. Each module can be viewed as a unit in control of one or several biological functions. As such, modules identify and bring together segments of a biological system. Gene network analysis has been used to detect gene modules associated with, for example, human diseases or plant seed germination, and to study the transcriptome landscapes and gene module organization in yeasts, plants, and animals [4–10].

Gene networks derived from microarray data have been described for plant species such as Arabidopsis, maize, and rice [6, 8, 11–13]. These networks have been constructed via a variety of approaches that employed different ways to measure interactions between genes. Most common are co-expression networks that utilize the Pearson correlation coefficient (PCC) to measure expression similarity between genes, where gene pairs with PPC larger than a chosen threshold value are considered to interact with each other. Examples included two Arabidopsis networks: one identified clusters of genes involved in processes such as photosynthesis, vitamin metabolism, or cell cycle-regulation, while a second network revealed groupings with cellular organelles and tissue-specific functions [8, 11]. Rice and maize networks were also assembled via the Weighted Gene Coexpression Network Analysis (WGCNA) method, which utilizes a power function of PCC to assess expression similarity [6, 12–14]. Specifically, Downs et al. used WGCNA to construct a maize developmental gene co-expression network that captured modules with tissue and developmental stages specificity, while Ficklin and Feltus compared WGCNA networks from maize and rice to identify conserved modules [6, 13].

Yet another way to approach co-expression network analysis uses the graphical Gaussian model (GGM), which utilizes partial correlation (Pcor) to identify association relationships between genes [15–17]. Significantly, Pcor determines the correlation that remains between two genes after removing the effects of all other genes. Pcor measures direct association between genes, while PCC often fails to differentiate between direct and indirect associations [15, 16]. Thus, Pcor is deemed as a better metric than PCC for gene network analysis [16, 18]. However, the utilization of GGM has been impeded by a requirement that the number of samples must be far larger than the number of genes. The original design was able to calculate Pcor data among a few thousand genes only [16]. Previously, we developed a random sampling-based method to overcome this obstacle and constructed the first genome-wide GGM network for Arabidopsis, followed by an updated network model termed AtGGM2014 [7, 17]. Compared to other networks, AtGGM2014 contained more genes and identified additional modules participating in a large variety of plant processes, like development, metabolism, response to stresses, and response to hormones [7]. For example, among many informative gene modules, it included hormonal signaling modules for phytohormones like auxin, abscisic acid, jasmonic acid, gibberellins, cytokinins, ethylene, and salicylic acid, demonstrating the network’s potential to facilitate systems biology studies on Arabidopsis gene functions.

More recently, a large collection of RNA-Seq based maize gene expression datasets have been generated by different groups and deposited in the public domain. Among these datasets are a gene expression atlas for 79 maize tissues [19, 20], as well as expression datasets for specific organs like inflorescences [21], leaves [22], ligules [23], embryos and endosperms [24], and transcriptome datasets for different compartments in the endosperm [25, 26]. Others resulted from monitoring maize responses to abiotic stresses [27, 28], fungal infections [29], and different nutrition regimes [30]. These datasets provide extremely valuable information for maize functional genomics research. A maize RNA-Seq-based gene network focused exclusively on development has been constructed from expression data of 23 different tissues, which identified 19 gene modules via the WGCNA method [31]. However, to our knowledge, missing so far is a comprehensive gene network analysis that combines the wealth of many different maize datasets and merges them into an inclusive network to outline future research opportunities.

Here, we constructed a maize GGM gene co-expression network using expression data from 787 RNA-Seq runs deposited in the NCBI SRA database. 964 gene modules were identified from this network, highlighting functions in various cell organization, development, nutrients, metabolism, and stress responses pathways. As examples, we describe in detail modules involved in the development of inflorescences, ligules, and kernels, the uptake and utilization of nitrogen and phosphate, the metabolism of benzoxazionids, oxylipins, flavonoids and wax, and the response to heat stress, endoplasmic reticulum (ER) stress, and fungal infections. These modules provide a general picture for the relevant biological processes and identified both known genes as well as potential, so far unconnected, candidate genes for future functional studies. Importantly, many of these modules contain transcription factor genes that act as potential gene expression regulator for the genes within the same modules. In addition, the maize network has been compared to a previous published Arabidopsis network [7]. This juxtaposition revealed conserved as well as diverging modules in the two species. The identified gene modules were further used to analyze a dataset on a maize leaf developmental series [22], demonstrating the usefulness of this network for systems biology analysis.

## Results

### Overview of the maize GGM gene network

**Fig. 1:**
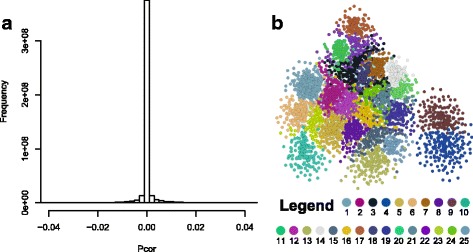
An overview of the maize GGM network. **a** A histogram showing the distribution of the partial correlation coefficients of all the gene pairs. Most genes pairs have their Pcors in the range between −0.01 and 0.01, indicating no interactions. **b** A sub-network for the largest 25 modules identified from the network. *Each dot* represents a gene, and a connection between two genes indicates interaction between the two. The *color of a node* indicates its module identity, as shown in the legend

A maize gene co-expression network based on GGM was constructed using maize RNA-Seq transcriptome datasets deposited in the NCBI Sequence Read Archive (SRA) database. The publicly available raw data files (.sra) were downloaded, mapped against the maize reference genome (AGP_v3.22), and processed into gene expression values. After removing files with mapping rates <70%, 787 RNA-Seq runs from 36 different studies (Additional file 1: Table S1) were retained and their Fragments Per Kilobase of transcript per Million mapped reads (FPKM)-normalized gene expression values were combined into a large gene expression matrix. These datasets monitor maize transcriptomes from various tissues and developmental stages, after a variety of biotic and abiotic stress treatments, or from well-characterized mutants. The genes with maximum FPKM values <20 were filtered out, resulting in a gene expression matrix that included 29,316 maize genes (rows) and 787 RNA-Seq runs (columns). Similar to other RNA-Seq data, these maize RNA-Seq data showed a mean-variance dependency [32], i.e. genes with higher mean expression values were more likely to have larger variances. The gene expression matrix was transformed via log-transformation to reduce such mean-variance dependency (Additional file 2: Fig. S1), making the dataset more suitable for correlation analysis [32]. Log-transformation has been used before for gene co-expression and gene clustering analysis with RNA-Seq data [33–40]. The log-transformed gene expression matrix was then used for the calculation of partial correlation coefficients (Pcor), following a procedure described before [17]. Figure 1a shows the distribution of the Pcors between all genes pairs, 98.4% of which are in the range between −0.01 and 0.01. The gene pairs with |Pcor| > = 0.035 (pValue = 2.22E-16) were kept for network construction. Additionally, Pearson Correlation Coefficients (PCC) was also calculated for all gene pairs and those with |PCC| < 0.35 were removed, reasoning that a low PCC very likely indicates independence between genes. As a result, 123,093 genes pairs with Pcor > = 0.035 and PCC > = 0.35 and 573 gene pairs with Pcor <= −0.035 and PCC < = −0.35 were selected for gene network construction (Additional file 3: Table S2).

This reasoning resulted in a maize GGM network including 20,269 genes and 123,666 co-expressed gene pairs (0.06% of all possible gene pairs). The clustering coefficient (*C*) of the network is 0.209, while the expected *C* of a random network of the same size is only 0.0006 [41]. Since *C* measures a network’s potential modularity [42], the large *C* of the maize GGM network indicates high characteristics of modularity. Indeed, a network clustering procedure using the MCL program [43] identified 964 gene modules that contain 5 or more genes each, and these 964 modules encompass 16,668 genes in total. These modules can be viewed as co-expression modules, whose genes share similar co-expression patterns and, according to the ‘guilt-by-association’ paradigm, might have similar functions or participate in the same pathways. The rest 3601 genes were assigned to small modules with 4 or less genes, which were not considered in further analysis. Within our network, the largest 222 modules containing between 15 and 306 genes (Fig. 1b, Additional file 4: Table S3). Based on Gene Ontology (GO) enrichment analysis, many of these modules fall into 5 distinct categories, with functions in: **i.** Cell organization; **ii.** Development; **iii,** Nutrient uptake & utilization; **iv.** Metabolism, including primary & secondary metabolism; and **v.** Stress responses (Table 1 & Additional file 1: Table S4). Modules for cell organization and primary metabolism include those for cell cycle regulation (Module #60), DNA replication (#64), cytoskeleton organization (#69), nucleosome assembly (#49), photosynthesis (#20 and 129), and mitochondria-related functions (#14 and 86) (Table 1). Several of these modules are highly conserved when compared to similar modules reported in a published Arabidopsis network, AtGGM2014 [7].

**Table 1:** Selected gene modules identified from the network

Category	Module no.	Number of genes within the module	Percentage of genes sharing conserved interactions with Arabidopsis	Module annotation	Selected genes from the module^a^	Enriched GO term	pValue
Cell Organization	49	71	80.28%	chromatin organization	his2a1/his2b1/his2b2/pcna2	nucleosome organization	2.15E-132
	60	59	44.07%	cell division	cyc1/hmg9/GRMZM2G061287(CYCB2;4)	cell division	1.32E-07
	64	58	63.79%	DNA replication	rfa1/rrb3/GRMZM2G139894(MCM2)	DNA replication	3.16E-39
	69	53	30.19%	cytoskeleton	krp2/krp4/krp8/cyc3/cyc8	microtubule motor activity	1.64E-46
Development	6	182	35.71%	seed maturation	ole1/ole3/vp1	monolayer-surrounded lipid storage body	1.75E-10
	16	139	12.95%	inflorescences development	ra1/ba1/ra3/spi1/tb1/bd1/yab10	abaxial cell fate specification	5.25E-10
	46	73	2.74%	kernels development	o2/pbf1/az19D2/az19D1/de30/zp1/zp22.1	nutrient reservoir activity	6.32E-64
	48	72	1.39%	ligules development	lg1/sbp28/ns1/myb43/sbp3	DNA binding	9.53E-06
	210	15	60.00%	ligules development	knox3/kn1/rs1/hb123/lg3/lg4/hb76/ hb8/hb48/gn1/knox5	sequence-specific DNA binding	8.77E-17
Nutrients	36	88	23.86%	response to phosphate starvations	ppck2/ppck3/ppck4/pht7	cellular response to phosphate starvation	9.62E-17
	72	51	43.14%	nitrate assimilation	nii2/nnr1/gln3/gln6	nitrate assimilation	1.18E-09
Primary Metabolism	14	151	18.54%	respiratory electron transport chain	GRMZM2G450825(COX1)/ GRMZM2G173833(COX2)	mitochondrion	9.10E-92
	20	130	69.23%	photosynthesis	cyb6/GRMZM2G448174(PETA)/ GRMZM2G433927(PSBE)	photosynthesis	5.46E-30
	86	40	42.50%	mitochondrial electron transport / ATP synthesis	nad1/nad4/GRMZM2G156068(ATP5)	mitochondrial membrane	9.35E-28
	129	26	65.38%	photosynthesis	gpa1/lhcb2/lhcb3/lhcb7	photosynthesis	1.69E-38
Secondary Metabolism	40	84	27.38%	wax biosynthesis	gl1/gl2/gl3/fdl1	wax biosynthetic process	2.25E-13
	65	57	29.82%	flavonoids biosynthesis	pr1/c2/whp1/chi1	flavonoid metabolic process	1.89E-12
	73	49	42.86%	fatty acid biosynthesis	wri1/wri2/acc1/acc2	monocarboxylic acid metabolic process	2.64E-27
	80	43	6.98%	benzoxazinoids and oxylipins biosynthesis	bx1/bx2/bx3/bx4/bx5/lox2/lox3/lox5/lox10	oxylipin metabolic process	6.12E-12
	154	20	30.00%	flavonoids biosynthesis	bz2/a1/bz1/a2/r1	flavonoid biosynthetic process	2.26E-08
Stress	5	188	15.96%	response to fungus	nactf7/chn1/chn2/sip1	defense response to fungus, incompatible interaction	3.27E-09
	10	157	47.13%	response to heat stress	hsp18c/hsp18f/hsp1/hsftf8/hsftf12	response to heat	8.75E-49
	95	35	65.71%	response to endoplasmic reticulum stress	bip1/bip2/der1/bzip60	response to endoplasmic reticulum stress	6.89E-15

^a^Shown in parenthesis are the maize genes’ homologues in Arabidopsis

**Fig. 2:**
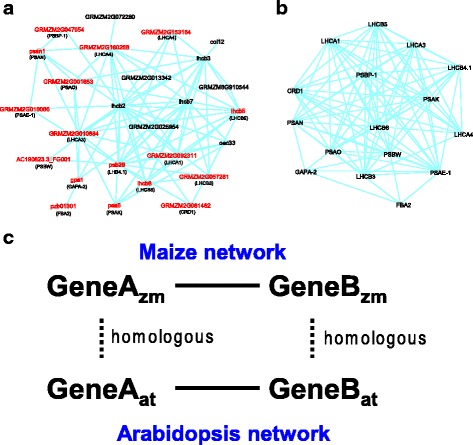
Network comparison between maize and Arabidopsis. **a** A maize module functioning in photosynthesis. *Red color* indicates the gene has conserved interaction(s) in the Arabidopsis network. The maize genes’ Arabidopsis homologues are shown in parenthesis. **b** An Arabidopsis sub-network for the homologues of the maize genes in (**a**). **c** A maize gene is considered to have conserved interaction(s) in Arabidopsis if its homologues and at least one of its interactors’ homologue also interact within the Arabidopsis network

Shown in Fig. 2a is **Module #129** for photosynthesis, and Fig. 2b is a subnetwork for Arabidopsis homologues of the genes in this module extracted from the Arabidopsis network AtGGM2014. The similarity of the structure and gene content in both species is significant. To enable direct comparison between the two networks, we consider a maize gene to have conserved interactions between the two networks if the maize gene and its Arabidopsis homologue share at least one homologous neighboring gene across the networks (Fig. 2c). According to this criteria, 9.7% of the genes in the whole maize network have conserved interactions. In contrast, 65% of the genes in Module #129 show conserved interactions in Arabidopsis, much higher than the network-wide level, indicating high conservation for the genes within this module. Within the modules for cell organization and mitochondria-related functions, 19% to 80% of the genes also revealed conserved interactions. We will describe modules in other GO categories with varied degrees of conservation between the two species.

### Gene modules revealing developmental features

**Fig. 3:**
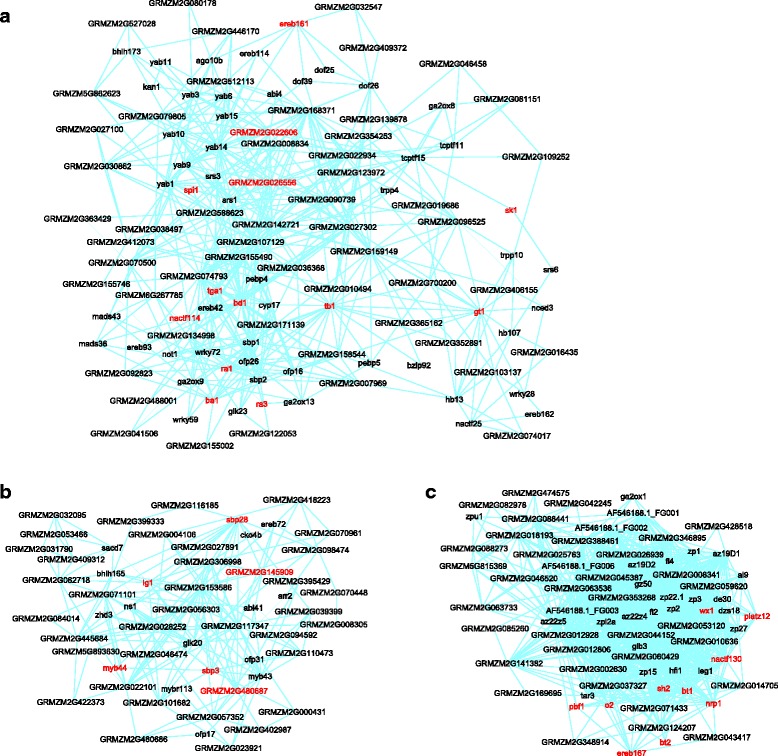
Three modules for the development of inflorescences (**a**), ligules (**b**), and kernels (**c**). Genes discussed in the text were highlighted in *red*. Due to the large sizes of the modules, only genes with 2 or more connections are shown

Maize plant architecture is a major yield-determining trait. Our gene network identified three particularly relevant modules. **Module #16** is enriched with genes involved in the development of ears and tassels, and contributes to shaping the maize inflorescences architecture (Fig. 3a, Table 1, and see Additional file 4: Table S3 for the complete list of genes within this module and the modules discussed below). According to published gene expression datasets on maize development [19–21, 23–26], the 139 genes within this modules are mainly expressed in ears, tassels, and immature cobs (Additional file 5: Fig. S2), and 56 of them encode transcription factors (TF). Included within the module are *ra1* (*ramosa1*) and *ba1* (*barren stalk1*), two TF genes with opposite roles in tassels development [44, 45]. Maize recessive mutants of *ra1* and *ba1* have increased and zero number of tassel branches respectively. A number of genes for the development of ears and tassels were included as well: *ra3* (*ramosa3*), *sk1* (*silkless ears1*), *spi1* (*sparse inflorescence1*), *tb1* (*teosinte branched1*), *gt1* (*grassy tillers1*), and *bd1* (*branched silkless1*), [46–51]. Also contained is *tga1* (*teosinte glume architecture1*), a gene conferring the naked kernel phenotype in maize [52]. Uncharacterized genes within the module include *GRMZM2G022606* and *GRMZM2G026556*, two genes homologous to *BOP2* in Arabidopsis. In Arabidopsis, *BOP1*, *BOP2*, and *PUCHI* (a *bd1* homologue) redundantly promote floral meristem fate [53], raising the possibility that the two maize genes might have a similar function. Also uncharacterized are *ereb161* and *nactf114*, homologous to Arabidopsis genes *ANT* and *CUC3* that regulate ovule and embryonic apical meristem formation, respectively [54, 55]. In addition, the module contains other potential development related genes as well, including 8 YABBY, 4 AP2/EREBP, 3 SHI RELATED SEQUENCE (SRS), and 3 SQUAMOSA PROMOTER BINDING PROTEIN (SBP) type TF genes. The genes in Module#16 could possibly form an elaborate yet balanced network orchestrating maize inflorescences development. And at least five of them, *ra1*, *ba1*, *gt1*, *tb1*, and *tga1*, have been subjected to selection during maize domestication and were designated as domestication genes [44, 45, 47, 51, 52, 56]. Interestingly, when compared to the Arabidopsis network, only 18 of the 139 genes within the maize module show conserved interaction(s), among them 7 YABBY TFs. Thus, only 8.4% of the genes revealed conserved interaction(s) when the YABBY genes are not considered. This indicates extensive pathways re-shuffling between the two species for inflorescences development.

Ligules are fringe-like tissues located at the junction of the leaf blade and leaf sheath. In maize ligules control leaf angles and affect vegetative architecture [57, 58]. Two modules involved in ligules development were identified. **Module #48** (Fig. 3b, Table 1 & Additional file 4: Table S3) is specifically expressed in the pre-ligule region of the leaf primordia (Additional file 5: Fig. S2). A key gene within the module is *lg1* (*liguleless1*), encoding a SBP TF that acts as a mater regulator of ligule development [58]. The recessive *lg1* mutations in maize erases ligules and renders leaves more upright compared to wild type leaves [57]. Interestingly, the genes within this module have their promoters enriched with a SBP TF binding motif “CGTAC” (pValue = 1.04E-6) [59], indicating they might be targets of Lg1. Two other SPB genes, *sbp3* and *sbp28*, are also included within the module, although their functions remain uncharacterized. Other potential development regulators included *myb44*, a homologue of Arabidopsis *LOF1* that functions in organ boundary specification [60], *GRMZM2G480687*, encoding a MEMBRANE-ASSOCIATED KINASE REGULATOR (MARK) proteins, and *GRMZM2G145909*, homologous to an Arabidopsis atypical bHLH gene *IBL1*. MARKs and atypical bHLH TFs in Arabidopsis participate in developmental processes mediated through brassinosteroids (BR) [61, 62], while BR-signaling also regulates ligules development in maize [63]. **Module #210** contains 15 genes, eight of which encode TFs in the KNOX family (Table 1 & Additional file 4: Table S3). Among these TFs are *gn1* (*gnarley1*), *kn1* (*knotted1*), *rs1* (*rough sheath1*), *lg3* (*liguleless3*), and *lg4* (*liguleless4*), none of which show any expression in the pre-ligule regions of the wild type plants (Additional file 5: Fig. S2). However, ectopic expression of any of these genes in their corresponding dominant mutant background affects and distorts leaf and ligule development [64–66]. It remains to be tested if the other 3 KNOX TFs within the module have similar functions and if and how these TFs function together to regulate ligules development.

Kernels development represents yet another critical process determining maize grain yield and quality. Five relevant gene modules are identified. Among them, **Module #46** (Fig. 3c, Table 1 & Additional file 4: Table S3), including 73 genes, is specifically expressed in the endosperm (Additional file 5: Fig. S2) and enriched with genes indicating nutrient reservoir activity (GO pValue = 6.32E-64). The module contains 16 genes encoding α-, δ-, or γ-zein proteins, the major seed storage proteins in maize, and 4 genes for starch biosynthesis (*bt1*, *bt2*, *sh2*, and *wx1*) [67, 68]. Notably, also included are a bZIP TF gene *o2* (*opaque endosperm2*) and a Dof TF gene *pbf1* (*prolamin-box binding factor1*), two master regulators of zein gene expression [69, 70]. It is recognized that within the maize network transcription factors and their target genes are often contained within the same modules, providing an edifying way to identify those modules’ expression regulator(s). Module #42 includes other TF genes as well, such as *ereb167*, *platz12*, *nrp1*, *nactf130*, as potential transcription regulators. **Module #6** is expressed in both embryo and endosperm tissues at late developmental stages (Additional file 5: Fig. S2) and enriched with genes functioning in seed maturation (pValue = 7.39E-6). The module includes many lipid storage genes (e.g. *oleosin1*), desiccation tolerance genes (e.g. genes encoding late embryogenesis abundant proteins), and the TF gene *vp1* (*viviparous1*), a master regulator of seed maturation and dormancy (Additional file 4: Table S3) [71]. 36% of the genes within this modules possess conserved interactions when compared with the Arabidopsis network, including the key seed development genes like *vp1*, *ole1*, *ole3*, and *mlg3*. Additional modules were recovered with specific expression in different compartments of the maize endosperm, such as the basal endosperm transfer layer (BETL) (#11), the embryo-surrounding region (ESR) (#32), and the placento-chalazal region (PC) (#53) (Additional file 6: Fig. S3), similar to a previous report [26]. These examples indicate that our network delineates different modules corresponding to different functional domains of kernels development and provides a general picture of the process.

Other modules were identified that draw attention to the development of other tissues and organs (Additional file 1: Table S4, Additional file 7: Fig. S4), such as anthers (#3, 17 and 22), meiotic tassels (#18), roots (#30), carpels (#92), Casparian strip (#76), and epidermal cells (#155). Furthermore, identified were modules for primary (#543) and secondary cell wall biosynthesis (#13), and for the signaling pathways of development related hormones, such as auxin (#158) and cytokinins (#183) (Additional file 1: Table S4). These modules are valuable resources for future functional studies on distinct and related developmental processes.

### Modules for nutrients uptake and utilization

**Fig. 4:**
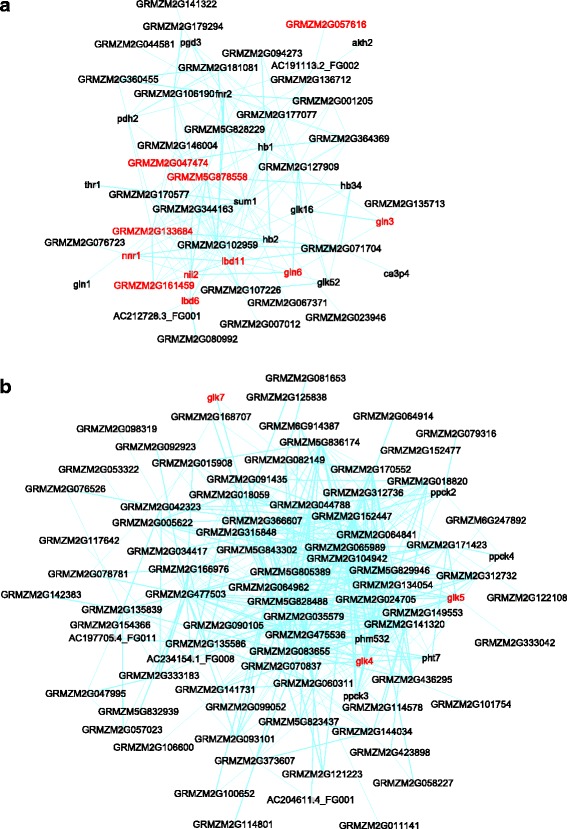
Modules for nitrogen uptake and utilization (**a**) and phosphate starvation response (**b**). Genes discussed in the text were highlighted in *red*

Nutrient use efficiency is a key objective for crop improvement. Modules can be pinpointed from the maize network as functioning in nitrogen, phosphate, iron, and sulfate uptake and utilization. In general, these modules are conserved between maize and Arabidopsis. For example, **Module #72** (Fig. 4a, Table 1 & Additional file 4: Table S3) shows enrichment for nitrate-responsive genes that encode the key enzymes for reducing and incorporating nitrate into glutamine, including nitrate reductases (*GRMZM5G878558* and *nnr1*), nitrite reductase (*nii2*), and glutamine synthetase (*gln1*, *gln3*, *gln6*) [72–74]. Also contained are *GRMZM2G161459* and *GRMZM2G057616*, homologues of the Arabidopsis genes *NRT1.1* (encoding a nitrate transporter and sensor) and *CLC-A* (encoding a vacuole nitrate transporter required for high nitrate uptake capacity) [75, 76]. Compared with the Arabidopsis network, 22 out of the 51 genes in this module showed conserved interaction(s). Among them are two unknown genes, *GRMZM2G047474* and *GRMZM2G133684*, whose homologues in Arabidopsis *AT5G39590* (encoding a TLD domain containing protein) and *AT5G62720* (encoding an integral membrane HPP family protein) are also included in an Arabidopsis nitrate-responsive module but they remain uncharacterized. Their appearance in nitrate responsive modules in both species strongly suggests functions in nitrate sensing, uptake and/or utilization. Also conserved are two uncharacterized LOB-type TF genes *lbd6* and *lbd11*, homologous to one Arabidopsis nitrate response regulator gene *LBD37* [77], indicating *lbd6* and *lbd11* arguably could have similar functions in maize. Additionally, the maize module includes genes encoding G2-like, HB, and C2H2 type TFs, whose functions in nitrate response have not yet been tested.

**Module #36** includes genes involved in the phosphate starvation response (pValue = 9.62E-17) (Fig. 4b, Table 1 & Additional file 4: Table S3). The maize module includes homologues of Arabidopsis phosphate starvation response genes, such as *SPX2*, *SPX3*, *SQD1*, *SQD2*, *MGD2*, and *PS2* [78], although their functions have not been characterized in maize. A P1BS motif “GNATATNC”, the binding site of the Arabidopsis G2-like transcription factor PHR1, a master regulator of phosphate starvation response gene expression [79], is enriched in the promoters of the genes in this module (pValue = 9.40E-24). Interestingly, the maize module contains three G2-like TFs, *glk4*, *glk5*, *glk7*, which could also act as master regulators.

Maize modules involved in the uptake and utilization of iron (#43) and sulfate (#79) were also identified (Additional file 1: Table S4). It should be noted that, compared to Arabidopsis, fewer transcriptome datasets are available for maize. As more data become available, more maize modules for nutrient uptake and usage should be revealed in future analysis.

### Modules for metabolic processes

**Fig. 5:**
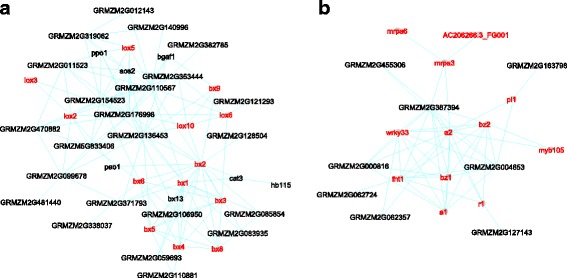
Modules for the biosynthesis of benzoxazinoids and oxylipins (**a**) and flavonoids (**b**). Genes discussed in the text were highlighted in *red*

Gene modules were identified for various processes in maize metabolism. In addition to those involved in primary metabolisms, numerous modules functioning in secondary metabolism emerge as well. Some of these modules are unique to maize, while others share considerable similarities between Arabidopsis and maize. Among them, genes in **Module #80** (Fig. 5a, Table 1 & Additional file 4: Table S3) provide functions in the production of benzoxazinoids and oxylipins, which are secondary metabolites effective in anti-herbivore defense [80]. Benzoxazinoids are mainly found in Poaceae species, including maize, wheat, and rye, but appear only infrequently in dicots and are absent in Arabidopsis [81]. Module #80 includes most known genes of the maize benzoxazinoids biosynthesis pathway – *bx1* (*benzoxazinless1*), *bx2*, *bx3*, *bx4*, *bx5*, *bx6*, and *bx8* that form a gene cluster in chromosome 4, and *bx9* in chromosome 1 [81]. Interestingly, none of these *bx* genes share any conserved interactions with the Arabidopsis network, consistent with the absence of benzoxazinoids biosynthesis pathway in Arabidopsis. Additionally, the module is enriched with oxylipins biosynthesis genes (pValue = 2.22E-10), such as *lox2*, *lox3*, *lox5*, *lox6*, and *lox10*. The inclusion of both benzoxazinoids and oxylipins biosynthesis genes implies this module’s function in defense response.

**Module #154** (Fig. 5b, Table 1 & Additional file 4: Table S3) is enriched with flavonoids biosynthesis pathway genes (pValue = 2.26E-08), including *fht1*, *a1*, *a2*, *bz1*, *bz2* [82]. The module also contains *pl1* (*purple plant1*) and *r1* (*colored1*)*,* two TF genes controlling anthocyanin biosynthesis [82], and two potential regulatory TFs *myb105* and *wrky33*. In Arabidopsis, *TTG2*, a homolog of *wrky33*, regulates tannin level in the seed coat [83], while the function of maize *wrky33* remains to be tested. The maize module also includes the gene *mrpa3*, encoding a tonoplast-localized anthocyanin transporter [84], and two uncharacterized transporter genes *mrpa6* and *AC206266.3_FG001*. In addition to Module #154, involvement in flavonoids biosynthesis is indicated for Module #65 as well.

Waxes, deposited on the aerial surface of plants as a water-proof layer, are essential for plants in that they not only significantly limit water loss, but also counteract environmental stresses [85]. **Module #40** (Additional file 8: Fig. S5, Table 1 & Additional file 4: Table S3) of the maize network is enriched with wax biosynthesis genes (pValue = 2.25E-13), i.e. *gl1*, *gl2*, and *gl3* [86]. Among the 84 genes within this module, 23 have conserved interactions when compared with the corresponding Arabidopsis network. Conservation extends to uncharacterized genes that are homologous to Arabidopsis wax biosynthesis genes like *KCS1*, *KCS6*, *KCS12*, *CER3*, and *LACS1* [86]. Another conserved gene pair are *fdl1* (*fused leaves1*) in maize and *MYB94* in Arabidopsis, homologous to each other, both identified recently as TF regulators of wax biosynthesis [87, 88]. The maize module also contains *gl3* (*glossy3*), another MYB TF as a master regulator of wax production [89]. Other potential TF regulators within the module include 3 AP-EREBP type TFs, *ereb12*, *ereb60*, and *ereb143*. Among them, *ereb60* shows homology to Arabidopsis *WRI1* that regulates the accumulation of fatty acids – precursors for wax biosynthesis [90]. Additionally, another related module for fatty acid biosynthesis (#73) was also identified from the network (Table 1).

In addition to benzoxazinoids, oxylipins, flavonoids, wax, and fatty acids, modules were also identified for the metabolism of suberin (#42), trehalose (#61), glucose (#234), glucan (#278), and lignins (#712) (Additional file 1: Table S4). Based on these modules, promising candidate genes can be selected for future functional studies.

### Modules for stress responses

Modules involved in abiotic stress responses were also identified. For example, the genes in **Module #10** (Additional file 8: Fig. S6, Table 1 & Additional file 4: Table S3) show relationships to heat stress responses. Enriched are heat shock stress response genes (pValue = 8.75E-49). 74 of the 157 genes within this module share conserved gene interaction(s) with an Arabidopsis heat shock related gene module [7]. The conserved genes include 6 heat shock transcription factor genes (*hsft7/8/12/20/24*) and a co-activator gene *MBF1C (GRMZM2G051135)*, highlighting their overarching importance in regulating heat activated gene expression regulation. The splicing regulator genes *SR45a (GRMZM2G073567)* and *SR30 (GRMZM2G331811)* are also included in the list of conserved genes, suggesting alternative splicing could play important roles in the heat shock response in both species. Similarly, **Module #95** (Additional file 8: Fig. S7, Table 1 & Additional file 4: Table S3) is enriched with ER stress response genes (pValue = 6.89E-15). 23 out of the 35 genes within the module shared conserved interactions with Arabidopsis, including *bip1*, *bip2*, *pdi1*, and *der1* [91]. Interestingly, contained within the maize module is a putative master regulatory TF gene *bzip60* [92], whose homologue in Arabidopsis, *bZIP60*, is a major regulator of the ER stress response [93], indicating *bzip60* might have similar function in maize.

Also identified from the network were modules related to biotic stress responses. For example, **Module #5** (Additional file 8: Fig. S8, Table 1 & Additional file 4: Table S3) is enriched with genes functioning in defense responses to fungal infections (pValue = 3.27E-09), such as *cta1*, *wip1*, *prp1*, and *tps6*. It contains a NAC type TF gene *nactf7*, a homologue of the Arabidopsis *NAC042* gene. In Arabidopsis, *NAC042* is a master TF that regulates the biosynthesis of the anti-fungal compound camalexin [94]. Although maize does not produce camalexin, the inclusion of *nactf7* in this anti-fungal module indicates that it may modulate other secondary metabolism processes to produce anti-fungal compounds. Indeed, within the module are many metabolism-related genes, including 9 genes encoding cytochrome P450 enzymes, whose roles in maize anti-fungal defenses remain to be studied. Interestingly, the module also encompasses genes for gibberellin biosynthesis (*ks1*, *ks4*, *ko2*, and *cpps2*), consistent with previous reports that infection by certain fungal pathogens upregulates gibberellin related genes in maize [95].

Additional defense related modules were identified from the network, for example Module #2 and #47 (Additional file 1: Table S4). These modules provide useful targets for future functional studies.

### Gene network comparison between maize and Arabidopsis

**Fig. 6:**
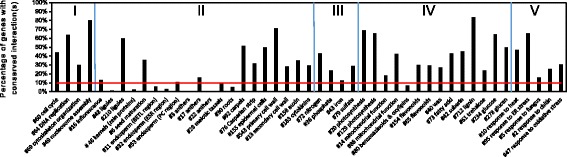
Network comparison between maize and Arabidopsis. For selected maize gene modules, the percentage of genes with conserved interaction in Arabidopsis were shown. Modules are organization by 5 categories: I, cell organization; II, development; III, nutrient uptake & utilization; IV, metabolism; V, stress response. The *red line* indicates the percentage of genes with conserved interaction(s) in the whole maize network

The examples discussed above demonstrate that some maize gene modules are conserved between maize and Arabidopsis, while others display greater divergence. The percentage of genes with conserved interaction(s) ranged from 0% to 83% for different modules (Fig. 6, Additional file 1: Table S5). Among them, the cell organization related modules retain the highest degree of conservation, in particular those for DNA replication and nucleosome assembly. Many modules in the nutrient uptake, stress-response, and metabolism categories display conservation above the network-wide average level, except for Module #80 with focus on benzoxazinoids and oxylipins biosynthesis. For the development modules, conservation varies significantly. Those involved in carpels, Casparian strip, epidermis, and cell wall development are conserved, all of which constitute the basic building blocks of the plant body. However, much more divergence is seen for modules for kernel/seed metabolism, anthers, inflorescences structure, or root development. Such difference can be attributed to the obvious biological differences between the two species, which define development, growth habitus and structure defining the two species. Nevertheless, both differences and similarities of genes identified in our high-stringency module structure can provide clues about unidentified functions in either one of the two species. The monocot/dicot comparison may further be exploited for additional gains in knowledge.

### Gene expression dynamics of gene modules in maize leaves

**Fig. 7:**
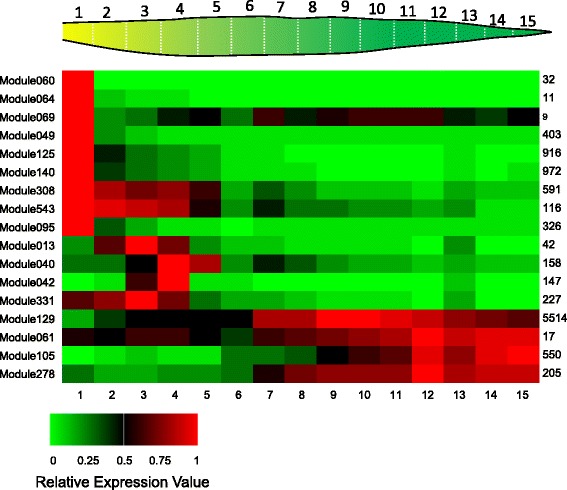
The average expression level for the genes within selected modules in the maize leaves transcription datasets. On *top* is a diagram of the 15 leaf segments. In the *middle* is a heatmap showing the relative expression level of each module in each segments compared to the highest expression level for that module over all segments, as indicated by the number on the right. A legend indicating the relative expression level is shown on the *bottom*

The maize gene modules identified from our network were used to re-analyze a previously published dataset that had measured transcriptomes from 15 segments of maize leaves, from the base to the tip, representing pronounced developmental gradients [22]. Owing to the pronounced differences in development and physiological functions in maize leaf segments, distinct expression dynamics are revealed in these modules. The average gene expression levels in each module for every segment was computed (Fig. 7). As expected, Module #60, involved in cell cycle regulation, is mainly expressed in Segment 1 at the base of the leaves, where cell division is most active. Other modules with peak expression at the base include those for DNA replication (#64), cytoskeleton organization (#69), nucleosome assembly (#49), ribosomal functions (#125, 140), plastid development (#308), primary cell wall biosynthesis (#543), and ER stress response (#95). These modules appear to be involved in the early development and building of leaf cells, and high expression of ER related genes might be indicators of intense protein synthesis. Peak gene expression for four modules is observed in the mid part of the leaves (Segments 2 to 5), including those for the biosynthesis of secondary cell walls (#13), waxes (#40), suberin (#42), and lignin (#331), likely representing maturation of the leaf tissue. Module #129, containing mainly light reaction-related photosynthesis genes, was highly expressed and peaked around Segments 9 and 10, which can be an indicator of overall light reaction intensity. Several modules functioning in carbohydrate metabolism or transport show peak expression at late stages (Segments 12 to 14), including #61 for trehalose biosynthesis, #278 for glucan metabolism, and #105 for carbohydrate transport (containing genes such as *sweet13a/b/c*). Thus, combining the modular arrangements revealed by the gene network with detailed leaf segment RNA-Seq datasets provided a coherent and detailed picture of maize leaf development, maturation, and biochemical activities. The results were consistent with the previous report [22, 96], and similar analysis can be applied to other maize datasets as well.

## Discussion

We report on the construction of a maize GGM gene co-expression network that includes 20,269 genes based on large-scale RNA-Seq transcriptome data. The resulting gene network was then analyzed and clustered via the MCL clustering algorithm [43]. Although the algorithm partitioned the network purely based on its topology, the analysis resulted in 964 distinct and informative gene modules that included functions in a wide range of maize physiological processes. These modules are particularly useful in that they can assign putative functions to unknown/uncharacterized genes and identify participating genes (including novel genes) for specific developmental or physiological processes, as demonstrated by the selected examples. Module structure and the nature of the genes assembled in a module may then be used to analyze individual gene expression datasets, for example the expression data on maize leaf segments.

The modules identified in our network analysis covered many aspects of maize biology. Compared to a previously published maize transcriptional network that contained 49 modules [6], our model defined 964 modules in total. Sizes of previously identified modules were large. Considering that 87% of all genes in previous network [6] were included in modules with more than 1000 genes it was difficult to pinpoint potential key regulatory genes as candidates for future studies. In contrast, our GGM network identified modules containing between 5 and 306 genes, which facilitated ranking of potentially interesting genes, as demonstrated by the presentation above for various modules. Also, we were particularly encouraged by the numerous examples in which individual GGM modules identified control genes that had previously been revealed and verified experimentally by mutants analysis, such as *ra1/3* in Module #16, *o2*, *pbf1* in Module #46, *bx1/2/3/4/5/6/8* in Module #80, and *pl1*, *r1* in Module #154 [44, 50, 69, 70, 81, 82]. In an additional contrast to Downs et al. (2013), where the focus on developmental microarray data sets recovered modules with tissue-specific expression, our maize GGM gene network is condition-independent, constructed from transcriptome datasets related to development, stresses, nutrition, as well as other treatments. This facilitated the distinction between modules involved in cell organization, development, nutrients utilization, metabolism, and stress responses. These modules could potentially play critical roles in determining important maize agronomic traits. For example, the inflorescences development related Module #16 includes 5 genes *ra1*, *ba1*, *gt1*, *tga1*, and *tb1* that have been shown by mutants analysis to regulate maize inflorescences architecture, and these 5 genes are designated as domestication genes since they were subjected to selections in the history of maize domestication as they control desirable traits [44, 45, 47, 51, 52, 56], while other genes within this module might also control maize architecture and performance. As well, modules for ligules and kernels development, for nitrogen and phosphate uptake and utilization, for primary and secondary metabolisms, and for responses to fungal infections include a wealth of information potentially useful for crop improvement.

An important feature of our network is that transcription factors and their target genes are often contained within the same module, suggesting shared expression characteristics. One example is the endosperm development Module #46, which is enriched with zein storage protein genes. The regulatory genes for zein biosynthesis, TF *o2* and *pbf1*, are contained within this modules as well. In yet another example, the master regulator TF of seed maturation, *vp1,* is included within the seed maturation Module #6. The ligule-related Module #48 contains the master regulator gene *lg1*, encoding a SPL TF. The genes within this module are enriched a SPL binding motif, indicating they are targets of Lg1. Similarly, the heat-induced Module #10 contains 6 heat shock TF genes *hsft7/8/12/20/24*, the ER-stress response Module #95 includes a putative maser regulator *bzip60*, and the phosphate starvation response Module #38 possesses three putative master regulator *glk4*, *glk5*, *glk7*. Significantly, many identified modules also included genes encoding unknown and uncharacterized TFs that may represent novel gene expression regulators. The modular structures, as revealed by our network, provide an expedient and edifying way to identify putative TF regulators for various maize pathways.

The maize gene network also enables cross-species comparison between maize and Arabidopsis. The comparison revealed an unexpected degree of insight into different degrees of conservation in different pathways. Not surprisingly, cell organization modules showed the highest percentage of conserved genes, indicating the evolutionary stability of such basic cellular pathways. Among development related modules, those involved in generating basic building blocks of the plant body are shown to be conserved as well, i.e. carpels, Casparian strip, epidermis, and cell walls. However, divergence was found for functions in modules related to the overall architecture of the plants, for example the development of inflorescence structures. Such comparison sheds light on pathways that might have been “hot targets” for evolutionary changes. In the near future, with RNA-Seq transcriptome datasets rapidly accumulating, such network comparison analysis can be extended into more plant species to identify steps that highlight and determine plant evolutionary trajectories. As shown here for maize and Arabidopsis, conversation and similarity in modular comparisons will assist in pinpointing key regulators in various modules that can then be analyzed in detailed studies.

## Conclusions

In conclusion, the maize GGM network presented here - in juxtaposition with a corresponding Arabidopsis network [7] - sheds light on similarities and differences in the organization of gene modules between different species in the context of evolutionary separation and different life histories. Additionally, our analysis highlights modules whose structure and gene content can provide important new resources for maize gene functional studies with application potential.

## Methods

### Maize RNA-Seq data collection

The publicly available maize RNA-Seq transcriptome datasets deposited in the NCBI SRA database were used in the analysis. These datasets were organized by studies. The studies were manually inspected to filter out those focusing on non-coding RNAs or those measuring transcriptome of the same tissues from a large number of maize varieties. Also removed were the studies with less than five RNA-Seq runs or without published articles. As a result, 36 studies were kept and their raw data files (sra) were downloaded. For RNA-Seq data processing, adapter sequences, if present, were removed from raw sequence reads using FASTX-toolkit pipeline version 0.0.13 (http://hannonlab.cshl.edu/fastx_toolkit/). Sequence quality was examined using FastQC (http://www.bioinformatics.babraham.ac.uk/projects/fastqc/), and low quality read was filtered by FASTX-toolkit. The remaining reads were then mapped to the maize genome AGP v3.22 (Ensembl Plants, http://plants.ensembl.org) using Tophat v2.0.10 [97] with default settings. After removing files with mapping rate smaller than 70%, the bam files from 787 RNA-Seq runs were analyzed to obtain gene expression values (FPKM) via Cufflinks v2.1.1 [98].

### Maize GGM network construction

The gene expression data were then merged into a single gene expression matrix with 787 columns, and the low expressed genes (maximum FPKM values among all samples being less than 20) were filtered out, resulting in a matrix with 29,316 genes and 787 columns. The matrix was log-transformed [32–40] via the log_2_ (FPKM + 1) function, a procedure that significantly reduced the dataset’s mean-variance dependency (Additional file 2: Fig. S1). The log-transformed gene expression matrix was then used for partial correlation calculation, following a method described before [17]. Briefly, the calculation involved a procedure with 25,000 iterations. In each iteration, 2000 genes were randomly selected and the partial correlation coefficients between gene pairs were estimated via the “ggm.estimate.pcor” function in the GeneNet v1.2.13 package in R [16]. The Pcors were recorded in every iteration. After 25,000 iterations, for every gene pair, the Pcor with the lowest absolute value was chosen as its final Pcor. The PCC between all gene pairs were also calculated. The gene pairs with Pcor > = 0.035 and PCC > = 0.35 and those with Pcor <= −0.035 and PCC < = −0.35 were selected for gene network construction (Additional file 2: Table S2), resulting in a maize GGM gene network based on the log-transformed gene expression data.

To evaluate the effect of log-transformation on the network quality, another gene network was also constructed directly from the gene expression matrix with the original FPKM values without data transformation, designated as non-transformed FPKM network, keeping all other parameters the same as in the log-transformation-based network outlined above. These two networks were then evaluated and compared via the EGAD package in R regarding their capacities to connect maize genes with shared GO terms [99]. The results indicated the log-transformation-based network out-performed the non-transformed FPKM network (Additional file 8: Fig. S9). Additionally, the log-transformation-based network identified gene modules that were not recovered by the non-transformed FPKM network, such as those related to ER stress response (#95) and nitrate response & assimilation (#72) (data not shown). These modules identified only by the log-transformation-based network contained genes that have been identified in different analyses to be related to the modules in question [73, 74, 91, 92].

Thus, we considered the higher power of the log-transformation-based network, and only results using the log-transformation-based network, designated as the Maize GGM Network, were further analyzed and discussed.

### Gene network properties and gene module identification

The R package of RBGL v 1.44.0 (http://bioconductor.org/packages/RBGL/) is used to calculate the clustering coefficient of the maize GGM network. The network was clustered via the MCL clustering algorithm, using these parameters “-I 1.5 -Scheme 7” [43]. The genes within each module were then analyzed for Gene Ontology enrichment via GOStats [100], with GO annotation file downloaded from the Gramene database (ftp://ftp.gramene.org/). The maize genes and their Arabidopsis homologues were further annotated with annotation files from MaizeGDB, TAIR, and PlnTFDB [101–103]. Selected modules were also tested for promoter motifs enrichment via the binomial distribution. An R script, included in the accompanied program MaizeGGM2016, was developed to extract sub-networks for gene modules and to draw development heatmaps for the genes within selected modules, with expression data from published datasets [19–21, 23–26]. The whole GGM network and the extracted sub-networks were layout and visualized with BioLayout Express 3D and Cytoscape 3.3, respectively [104, 105].

### Gene network comparison between the maize network and the Arabidopsis network

To enable comparison between the maize GGM network and the Arabidopsis network AtGGM2014, the InParanoid program (v 4.1) [106] was used to identify the maize genes’, if present, most similar homologues in Arabidopsis. For any gene within the maize network, if there exists a homologous gene within the Arabidopsis AtGGM2014 network, the maize gene’s immediate neighboring genes within the maize network were extracted as group A. Also extracted, as group B, were its homologous gene’s neighbors within the Arabidopsis network. If any of the gene within group A has a homologous gene in group B, the original maize gene was considered to have conserved interaction within the Arabidopsis network. For any given module, the percentage of genes with conserved interaction was calculated as an indicator of evolution conserveness or divergence.

## Additional files

Additional file 1: Table S1. The RNA-Seq studies used in the analysis. **Table S4.** Selected gene modules identified from the network. **Table S5.** The percentage of genes with conserved interactions within each module. (XLSX 45 kb)

Additional file 2: Figure S1. Log-transformation reduced the mean-variance dependency of the maize RNA-Seq data. **a** The standard deviation of each gene’s non-transformed FPKM expression values across all 787 RNA-seq runs are shown against the rank of genes. Genes are ranked by their mean expression values, from low (left) to high (right). The red line depicts a trend line of standard deviation, which indicates a clear mean-variance dependency. **b** The standard deviation of each gene’s expression values after log transformation across all RNA-Seq runs against the rank of genes. The mean-variance dependency is greatly reduced. (PDF 5820 kb)

Additional file 3: Table S2. The 123,666 gene pairs used for the maize GGM gene network construction. (TXT 4378 kb)

Additional file 4: Table S3. The 964 gene modules identified from the network. Listed are the genes’ names and their module identity, and the results of gene ontology enrichment analysis. (XLSX 3251 kb)

Additional file 5: Figure S2. A heatmap showing the tissue-specific gene expression patterns for the inflorescences, ligules, and kernels development related modules. The data source of the SRA studies were labeled in the sample names. Listed are the maize genes names and its symbol in lowercase letters, or, if it has no symbols, the names of its Arabidopsis homologues. (PDF 2927 kb)

Additional file 6: Figure S3. A heatmap for modules related to endosperm development. (PDF 2005 kb)

Additional file 7: Figure S4. A heatmap for additional modules related to development. (PDF 4276 kb)

Additional file 8: Figure S5. A module for wax biosynthesis. **Figure S6.** A module for heat-shock stress response. **Figure S7.** A module for ER-stress response. **Figure S8.** A module for stress response to fungus. **Figure S9.** Comparison between the non-transformed FPKM network and the log-transformation-based network. Both networks were evaluated via the EGAD package [99] in R regarding their capacities to connect genes with shared GO terms. For each GO term, the maize genes with that GO were considered as a gene set, and an AUROC value was calculated for each network using the EGAD package. A higher AUROC value indicates genes within that gene set are more likely to have each other as neighbors, and thus a better performance of the network. The histogram shows the overall distribution of the AUROC values for 1728 GO terms calculated for the non-transformed FPKM network (green bar) and for the log-transformation-based network (transparent bar with black border). The log-transformation-based network has more GO terms with higher AUROC values, thus it performs better than the other network. (PDF 795 kb)
