# Supplementary figures and images for: Maize network analysis revealed gene modules involved in development, nutrients utilization, metabolism, and stress response
Source: BMC Plant Biol. 2017 Aug 1;17:131. doi: 10.1186/s12870-017-1077-4 (PMC5540570; doi:10.1186/s12870-017-1077-4)

# Figure S1

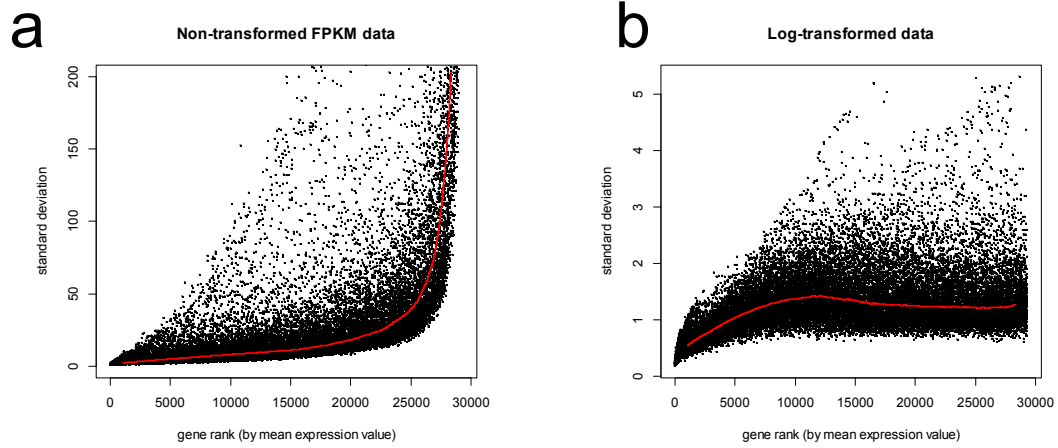

Supplement: Supplementary file 2 — Log-transformation reduced the mean-variance dependency of the maize RNA-Seq data. a The standard deviation of each gene’s non-transformed FPKM expression values across all 787 RNA-seq runs are shown against the rank of genes. Genes are ranked by their mean expression values, from low (left) to high (right). The red line depicts a trend line of standard deviation, which indicates a clear mean-variance dependency. b The standard deviation of each gene’s expression values after log transformation across all RNA-Seq runs against the rank of genes. The mean-variance dependency is greatly reduced. (PDF 5820 kb) [file 12870_2017_1077_MOESM2_ESM.pdf]

## Figure S2

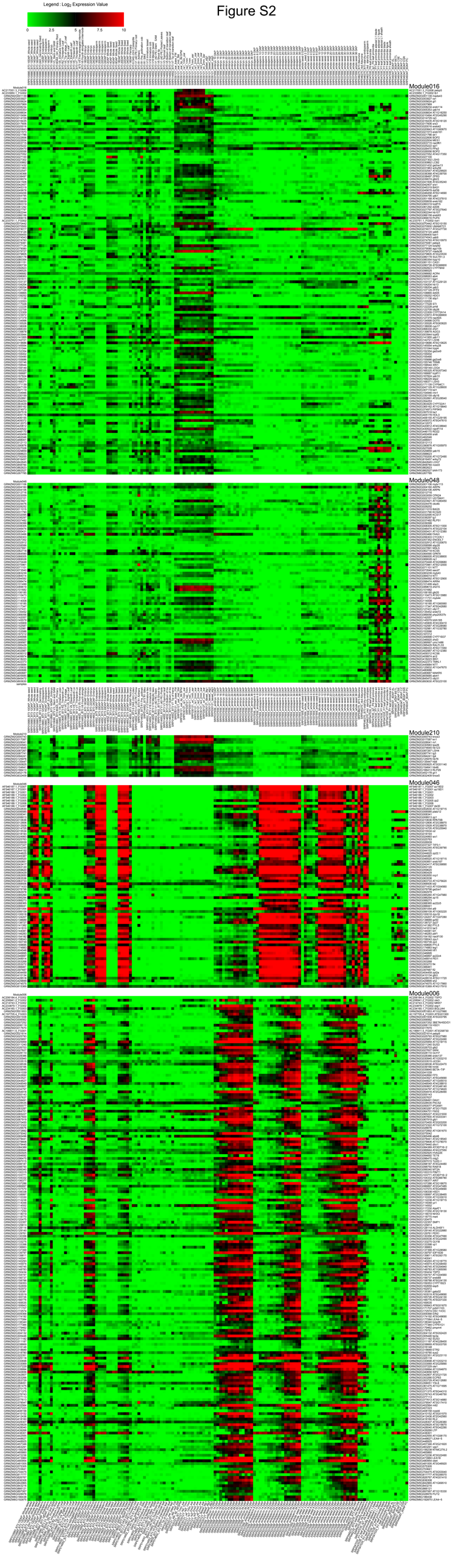

Supplement: Supplementary file 5 — A heatmap showing the tissue-specific gene expression patterns for the inflorescences, ligules, and kernels development related modules. The data source of the SRA studies were labeled in the sample names. Listed are the maize genes names and its symbol in lowercase letters, or, if it has no symbols, the names of its Arabidopsis homologues. (PDF 2927 kb) [file 12870_2017_1077_MOESM5_ESM.pdf]

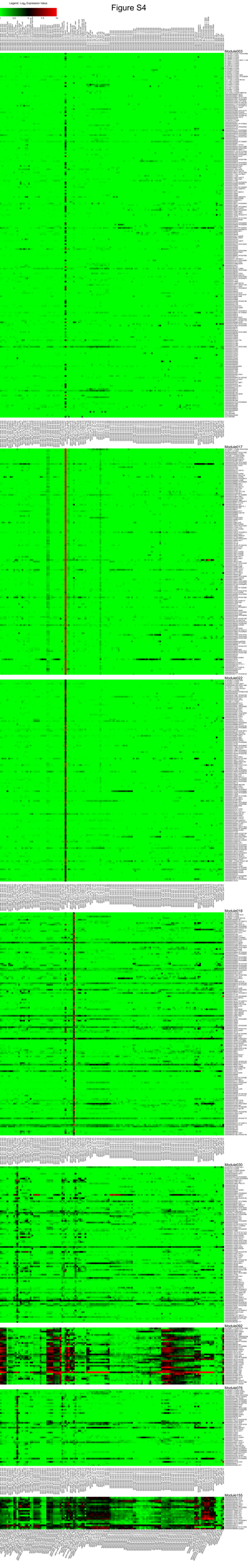

Supplement: Supplementary file 7 — A heatmap for additional modules related to development. (PDF 4276 kb) [file 12870_2017_1077_MOESM7_ESM.pdf]

# Figure S5

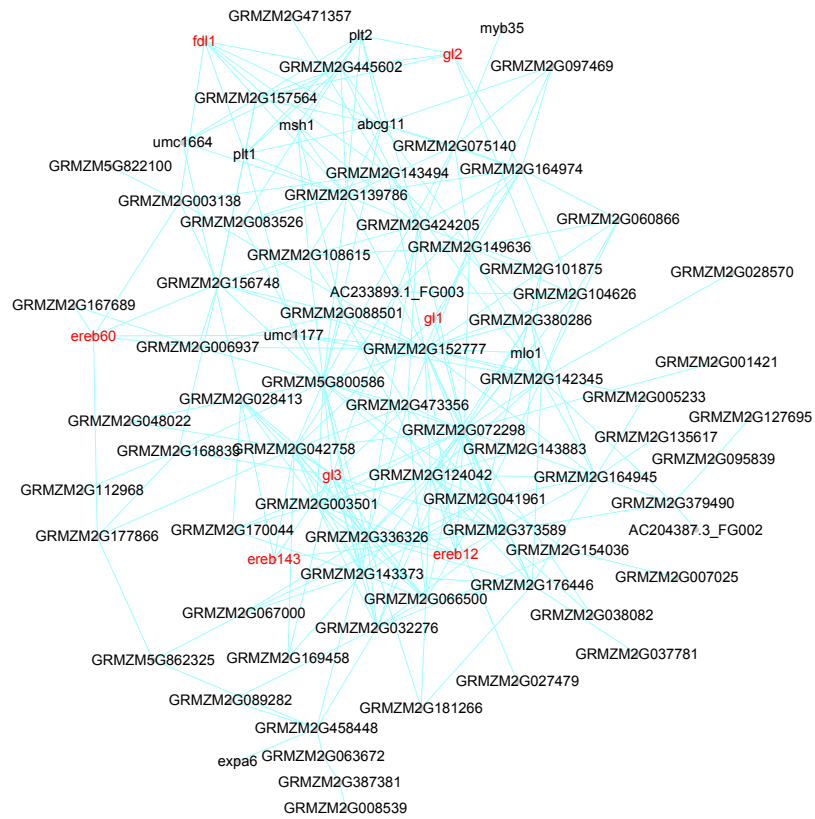



# Figure S7

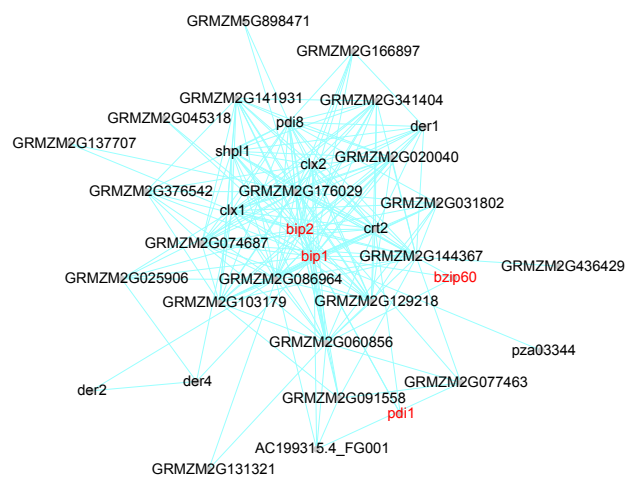



# Figure S9

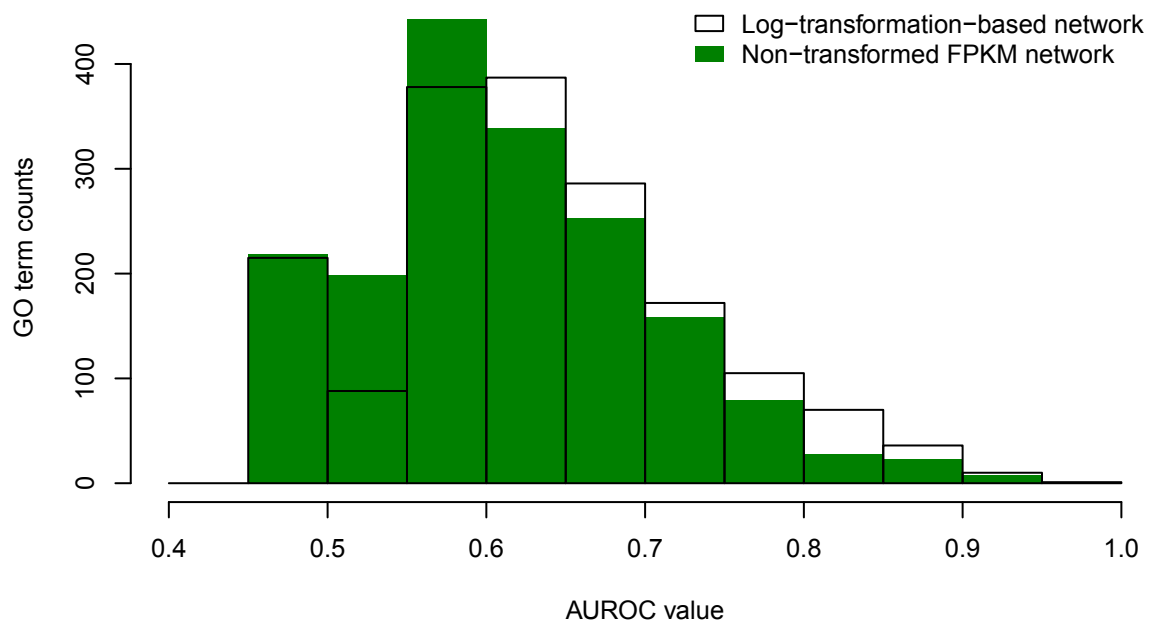

Supplement: Supplementary file 8 — A module for wax biosynthesis. Figure S6. A module for heat-shock stress response. Figure S7. A module for ER-stress response. Figure S8. A module for stress response to fungus. Figure S9. Comparison between the non-transformed FPKM network and the log-transformation-based network. Both networks were evaluated via the EGAD package [99] in R regarding their capacities to connect genes with shared GO terms. For each GO term, the maize genes with that GO were considered as a gene set, and an AUROC value was calculated for each network using the EGAD package. A higher AUROC value indicates genes within that gene set are more likely to have each other as neighbors, and thus a better performance of the network. The histogram shows the overall distribution of the AUROC values for 1728 GO terms calculated for the non-transformed FPKM network (green bar) and for the log-transformation-based network (transparent bar with black border). The log-transformation-based network has more GO terms with higher AUROC values, thus it performs better than the other network. (PDF 795 kb) [file 12870_2017_1077_MOESM8_ESM.pdf]
