# Supplementary material for: Maize network analysis revealed gene modules involved in development, nutrients utilization, metabolism, and stress response
Source: BMC Plant Biol. 2017 Aug 1;17:131. doi: 10.1186/s12870-017-1077-4 (PMC5540570; doi:10.1186/s12870-017-1077-4)
Supplement: Supplementary file 6 — A heatmap for modules related to endosperm development. (PDF 2005 kb) [file 12870_2017_1077_MOESM6_ESM.pdf]

Figure S3

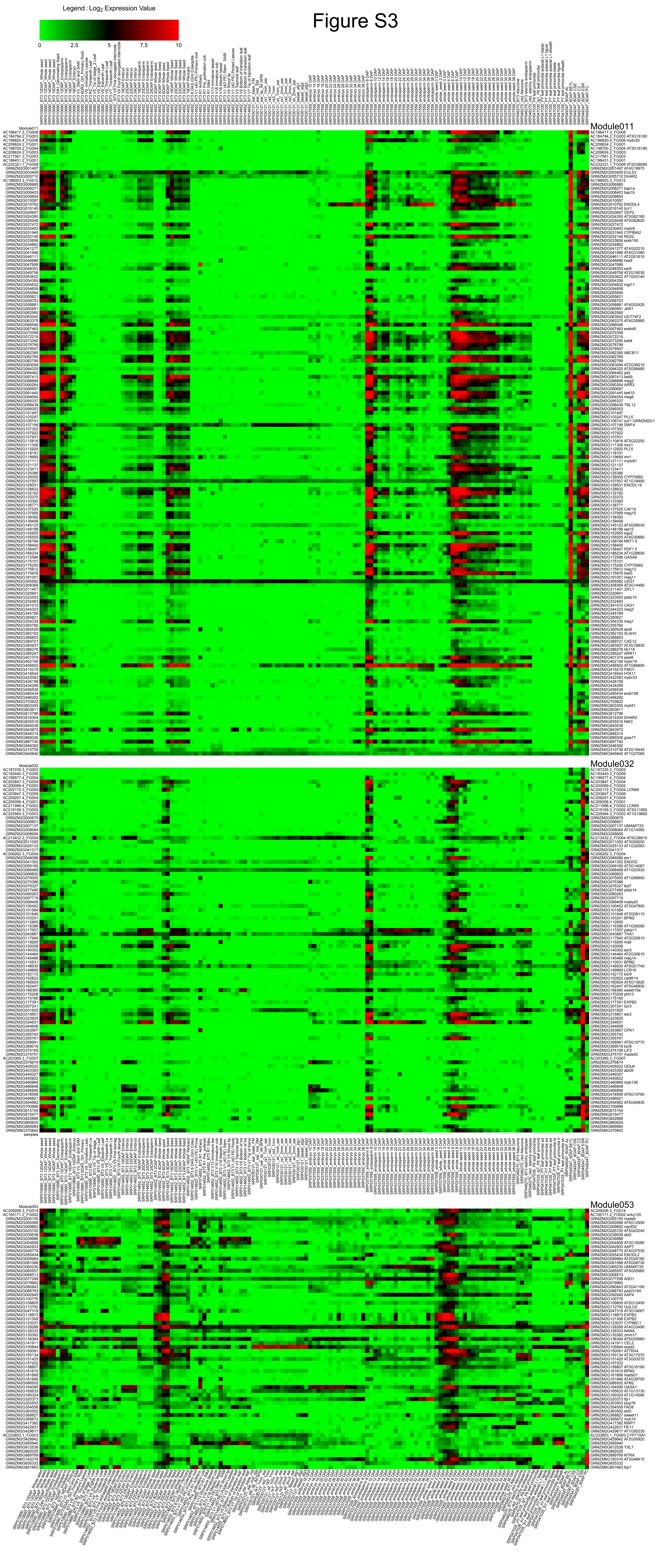

Legend : Log2 Expression Value
0
2.5
5
7.5
10
Module011
Module032
Module053
